# Os Biomarcadores podem ser Utilizados para Prever Recorrência de Arritmia após a Ablação de Fibrilação Atrial Guiada pelo *Ablation-Index* ?

**DOI:** 10.36660/abc.20230544

**Published:** 2024-04-15

**Authors:** Andreia Palma, Pedro A. Sousa, Carolina Saleiro, Sérgio Barra, Natália António, Luis Adão, João Primo, Ana Lebreiro, Paulo Fonseca, Luís Elvas, Lino Gonçalves

**Affiliations:** 1 Hospital Pediátrico de Coimbra Coimbra Portugal Hospital Pediátrico de Coimbra, Coimbra – Portugal; 2 Centro Hospitalar e Universidade de Coimbra Departamento de Eletrofisiologia e Estimulação Coimbra Portugal Departamento de Eletrofisiologia e Estimulação – Centro Hospitalar e Universidade de Coimbra, Coimbra – Portugal; 3 Hospital da Luz Arrábida Vila Nova de Gaia Portugal Hospital da Luz Arrábida, Vila Nova de Gaia – Portugal; 4 Universitário São João Departamento de Cardiologia do Centro Hospitalar Porto Portugal Departamento de Cardiologia do Centro Hospitalar e Universitário São João, Porto – Portugal; 5 Vila Nova de Gaia e Espinho Hospital Departamento de Cardiologia Vila Nova de Gaia Portugal Departamento de Cardiologia – Vila Nova de Gaia e Espinho Hospital, Vila Nova de Gaia – Portugal

**Keywords:** Biomarcadores, Ablação por Cateter, Fibrilação Atrial, Arritmias Cardíacas

## Abstract

**Fundamento:**

O software *ablation index* (AI) permitiu melhorar os resultados da ablação de fibrilação atrial (FA), mas as taxas de recorrência permanecem significativas. Biomarcadores séricos específicos têm sido associados a essa recorrência.

**Objetivos:**

Avaliar se certos biomarcadores podem ser utilizados (individualmente ou combinados) para predizer a recorrência de FA pós ablação guiada pelo AI.

**Métodos:**

Estudo multicêntrico, observacional, prospectivo de pacientes consecutivos, encaminhados para ablação de FA de janeiro de 2018 a março de 2021. Hemoglobina, peptídeo natriurético cerebral (BNP), proteína C reativa, troponina I ultrassensível, *clearance* de creatinina, Hormônio Tireoestimulante (TSH), e Tiroxina livre (T_4_) foram avaliados quanto à capacidade de prever a recorrência de arritmias durante o acompanhamento. Valores de p <0,05 foram aceitos como estatisticamente significativos.

**Resultados:**

Um total de 593 pacientes foram incluídos – 412 com FA paroxística e 181 com FA persistente. Durante o seguimento médio de 24±6 meses, 76,4% não apresentaram recidiva após ablação. Individualmente, os biomarcadores demonstraram um valor preditivo baixo ou nulo para recorrência. No entanto, TSH >1,8 μUI/mL [HR=1,82 (IC95%, 1,89-2,80), p=0,006] foi um preditor independente de recorrência. Avaliando-se a combinação de TSH, FT_4_ e BNP, a adição de cada valor “anormal” foi associada a uma menor sobrevida livre de recorrência (87,1% se nenhum vs. 83,5% se um vs. 75,1% se dois vs. 43,3% se três biomarcadores, p<0,001). Doentes com três biomarcadores “anormais” apresentaram três vezes maior probabilidade de recorrência de FA, comparativamente aos que não apresentaram nenhum biomarcador “anormal” (HR=2,88 [IC95%, 1,39-5,17], p=0,003).

**Conclusões:**

Quando combinados, valores anormais de TSH, FT_4_ e BNP podem ser uma ferramenta útil para prever a recorrência de FA pós ablação guiada pelo AI.

**Figure:**
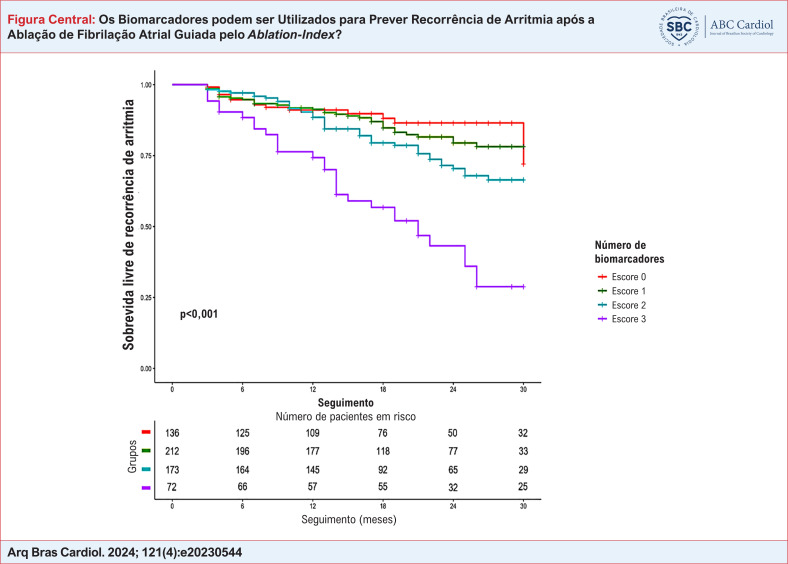
Curvas de Kaplan-Meier para sobrevida livre de recorrência de arritmia, segundo o número biomarcadores em níveis “anormais” (87,1 % para nenhum biomarcador vs. 83,5% para um vs. 75,1% para dois biomarcadores vs. 43,3% para três biomarcadores, p<0,001).

## Introdução

A fibrilação atrial (FA) é a arritmia cardíaca sustentada mais comum e causa de elevados custos sociais e de saúde devido à utilização recorrente dos serviços de saúde para o manejo de sintomas e pela morbidade associada.^1 , 2^ A prevalência da FA aumenta com a idade, variando de 0,5% em indivíduos com idade entre 50 e 59 anos a 8,8% em indivíduos com idade entre 80 e 89 anos.^3^ A ablação por radiofrequência (RF) surgiu como opção terapêutica para a FA, mas apesar dos avanços recentes, as taxas de recorrência continuam consideravelmente altas.^4^ Recentemente, um novo *software* intitulado “ *Ablation Index* ” (AI, ou “Índice de Ablação”, Biosense Webster), que incorpora força de contato, tempo e potência em uma fórmula ponderada, tem sido associado com menor ocorrência de reconexão da veia pulmonar, e sobrevida livre de arritmias, variando de 78% a mais de 90%.^5 – 9^

Identificar um maior risco de recorrência de arritmia pode ajudar médicos a selecionar pacientes para ablação, informá-los sore os riscos e benefícios, e selecionar a melhor estratégia de ablação. Vários biomarcadores foram associados com recorrência de arritmia atrial após a ablação da FA. Porém, os resultados variaram substancialmente entre os estudos, e o valor preditivo desses biomarcadores foi baixo.^10 – 18^ A maioria desses estudos avaliou o impacto dos biomarcadores individualmente em vez de inclui-los em um escore multiparamétrico. Além disso, com exceção de um estudo,^19^ nenhum biomarcador foi avaliado no contexto da ablação guiada por um software desenvolvido para predizer lesão transmural.

O objetivo deste estudo foi identificar biomarcadores séricos pré-ablação, associados com recorrência de arritmia, no contexto da ablação da FA guiada pelo software AI.

## Métodos

### Delineamento e local do estudo

Estudo observacional, prospectivo, multicêntrico de pacientes consecutivos encaminhados para ablação da FA entre janeiro de 2018 e março de 2021. Pacientes com FA paroxística ou persistente, encaminhados para ablação por cateter, foram submetidos a um protocolo específico de ablação. Dados clínicos basais e parâmetros da ablação foram obtidos dos bancos de dados do hospital. Valores séricos dos biomarcadores pré-ablação foram avaliados quanto à sua associação ou não com recorrência de arritma durante o acompanhamento.

Todos os pacientes assinaram um termo de consentimento, e o estudo foi aprovado pelo comitê de ética institucional local.

### Critérios de eligibilidade dos pacientes

Os pacientes foram elegíveis para inclusão no estudo se preenchessem os seguintes critérios de inclusão: 1) pacientes com FA paroxística, persistente ou persistente de longa duração, com idade igual ou superior a 18 anos, refratários ou intolerantes à terapia com Drogas Antiarrítmicas (DAA), 2) pacientes submetidos à ablação por RF utilizando um cateter irrigado para ablação por força de contato, guiada pelo software AI. A FA paroxística foi definida como FA com término espontâneo ou tratada com cardioversão em sete dias; FA persistente foi definida como FA sustentada por mais de sete dias ou tratada com cardioversão após sete ou mais dias; e FA persistente de longa duração foi definida como FA contínua por um ano e com estratégia de controle do ritmo, de acordo com as diretrizes da Sociedade Europeia de Cardiologia de 2020, elaborada com a colaboração da Associação Europeia de Ritmo Cardíaco.^20^

Os critérios de exclusão foram: história de ablação da FA ou síndrome coronariana aguda clinicamente aparente, contraindicação para anticoagulação e presença de trombo intracardíaco detectado antes do procedimento de ablação. Hipertireoidismo e hipotiroidismo foram considerados contraindicação para ablação por cateter.

História conhecida de doença tireoidiana foi definida como história de tireoidite, tireoidectomia, ou em tratamento clínico para hipertireoidismo ou hipotiroidismo, independentemente dos níveis atuais de tiroxina (T4) livre.

### Procedimento de ablação

Detalhes do manejo periprocedural e técnicas individualizadas de ablação de FA persistente e paroxística realizados em nossa instituição foram publicados anteriormente,^21 – 23^ e descritos detalhadamente no material suplementar.

### Medidas dos biomarcadores

Todos os biomarcadores séricos – Hemoglobina (Hb), Peptídeo Natriurético Cerebral (BNP), Proteína C-reativa (PCR), Troponina Ultrassensível (Tn-us), *Clearance* de Creatinina (Cl Cr), Hormônio Tireoestimulante (TSH) e Tiroxina (T_4_) livre – foram medidos até 18 horas antes do procedimento (independente da frequência cardíaca), com o paciente em posição supina, seguindo-se protocolos locais. Para a medida da Tn-us sérica (expressa em ng/L), o plasma foi separado por centrifugação a 3500 rpm por 15 minutos e medido logo em seguida. Os níveis de Tn-us foram analisados usando o teste diagnóstico Alinity^®^ (Abbot). O ponto de corte para o percentil 99 da Tn-us foi 16 ng/L. Os níveis plasmáticos de PCR foram medidos por um imunoensaio em látex (turbidimetria) (Alinity c CRP Vario, *Abbott Diagnostics* ). Os valores de referência para PCR são abaixo de 0,5mg/dL, sendo o limite inferior de detecção pelo teste de 0,1 mg/dL, e o limite mais alto 48mg/dL. Os níveis de BNP (pg/mL) foram medidos com um autoanalisador (Alinity, Abbot Diagnostics) usando o imunoensaio de micropartículas por quimioluminescência. O ponto de corte considerado normal foi <100pg/mL. Valores séricos de TSH e T4 livre foram medidos por imunoensaio de micropartículas por quimioluminescência (Alinity, Abbott Diagnostics). Os valores laboratoriais de referência para T_4_ livre e TSH foram 0,7 a 1,5 ng/dL e 0,4 a 4,0 μUI/mL, respectivamente. A creatinina sérica foi avaliada com um imunoensaio comercial com látex (Alinity, Abbott Diagnostics). Os níveis de creatinina (mg/dL) considerados normais foram entre 0,55 e 1,02 mg/dL (mulheres) e entre 0,72 e 1,18 mg/dL (homens). O Cl Cr foi calculado usando a equação de Cockcroft-Gault. Os níveis de Hb (g/dL) foram medidos por fotometria, utilizando um sistema de hematologia automático (Sysmex XN-9000, Sysmex) e o método SLS-Hgb (lauril sulfato de sódio, livre de cianeto). Entre os homens, os níveis de Hb entre 13,5 e 17,5 g/dL (18 a 49 anos de idade), 12,0 e 15,6 g/dL (49 a 65 anos) e entre 11,8 e 15,8 g/dL (>65 anos de idade) foram considerados normais. Nas mulheres, os valores de corte para Hb foram de 12,0 a 16,0g/dL (18 a 49 anos de idade), 12,0 a 15,6 g/dL (49 a 65 anos de idade) e de 11,8 a 15,8 g/dL (>65 anos).

### Desfechos do estudo

O objetivo primário foi avaliar se biomarcadores séricos, isolados ou combinados, podem ser usados para predizer a recorrência de arritmia após a ablação da FA guiada pelo AI. A recorrência de arritmia foi definida como a documentação de pelo menos 30 segundos de arritmia atrial sustentada após um período de três meses ( *blanking* ), independentemente dos sintomas.^24^

### Acompanhamento

Após o procedimento índice, os pacientes foram acompanhados por um mínimo de 12 meses. Os pacientes foram avaliados antes da alta, e aos três, seis, 12, 18 e 24 meses após o procedimento. A ecocardiografia transtorácica e o monitoramento por Holter 24 horas foram realizados antes da alta. As informações coletadas durante o seguimento incluíram um eletrocardiograma de 12 derivações e um Holter 24 horas em cada visita. Um monitoramento por Holter por sete dias foi realizado no mínimo uma vez ao ano. Na alta, o uso de DAA foi interrompido nos pacientes com FA (exceto os betabloqueadores). Nos pacientes com FA persistente ou persistente de longa duração, a prescrição de DAA ficou à critério do médico. Os primeiros três meses após o procedimento foram considerados como um período de *blanking* , e recorrências após esse período não foram consideradas. A estratégia de anticoagulação após os três primeiros meses foi baseada no escore CHA2DS2Vasc.

### Análise estatística

A análise estatística foi realizada usando o programa IBM SPSS Statistics versão 25 (IBM, Armonk, Nova Iorque) e o programa MedCalc Ltd. As variáveis categóricas foram expressas como frequências e porcentagens, e as variáveis contínuas com e sem distribuição normal como média ± desvio padrão e mediana e Intervalo Interquartil (IIQ), respectivamente. O teste X^2^ foi usado para avaliar diferenças ente variáveis categóricas e o teste t de Student e o teste Mann-Whitney-Wilcoxon foram usados para comparar as variáveis contínuas com e sem distribuição normal, respectivamente. O teste de Kolmogorov-Smirnov foi usado para testar a normalidade da distribuição das variáveis contínuas. A área sob a curva ROC (AUC) foi usada para testar o desempenho discriminatório de cada biomarcador, ou de sua combinação, na predição da recorrência de arritmia. Para cada preditor, o valor com a melhor sensibilidade e a melhor especificidade foi definido de acordo com o Índice de Youden. Esse valor foi usado para dicotomizar os biomarcadores séricos como “normais” ou “anormais” e como uma variável dicotômica na análise de sobrevida. As curvas de Kaplan-Meier foram criadas para ilustrar a sobrevida livre de arritmia de acordo com as diferentes combinações de biomarcadores. Um modelo de riscos proporcionais de Cox com covariáveis dependentes de tempo para a mudança da combinação de biomarcadores e recorrência de FA foi construído para avaliar o efeito independente dessas combinações sobre os desfechos. Todas as variáveis demográficas, clínicas e laboratoriais consideradas a exercerem um impacto sobre a recorrência de FA foram testadas em análise univariada. As variáveis que atingiram significância estatística foram incluídas no modelo de análise multivariada. Além disso, realizou-se uma sub-análise para os pacientes com FA paroxística e pacientes com FA persistente. Valores de p inferiores a 0,05 foram considerados estatisticamente significativos.

## Resultados

Dos 705 pacientes inicialmente submetidos à ablação da FA durante o período de recrutamento, 98 foram excluídos devido à história prévia de ablação da FA e 14 pacientes foram perdidos durante o seguimento (Figura S-1). A amostra final incluiu 593 pacientes, correspondendo a 412 pacientes com FA paroxística e 181 pacientes com FA persistente e FA persistente de longa duração. Várias diferenças nas características basais foram encontradas entre os pacientes com FA paroxística e os pacientes com FA persistente ( Tabela 1 ). Não foram observadas diferenças quanto à existência de doença tireoidiana conhecida ou ao tratamento prévio com amiodarona.

**Tabela 1:** Características basais dos pacientes com fibrilação atrial

	Todos os pacientes (n=593)	FA paroxística (n=412)	FA persistente (n=181)	Valor p
Sexo masculino, n (%)	345 (58)	234 (57)	111 (61)	0,30
Idade, anos (média ± DP)	59 ± 12	58 ± 12	60 ± 13	0,07
IMC, Kg/m^2^ (média ± DP)	27 ± 5	27 ± 5	27 ± 4	0,59
Hipertensão, n (%)	235 (40)	244 (59)	113 (62)	0,48
Diabetes mellitus, n (%)	376 (65)	268 (67)	108 (61)	0,16
História de AVC, n (%)	27 (5)	18 (4)	9 (5)	0,75
Insuficiência cardíaca congestiva, n (%)	132 (23)	60 (15)	71 (41)	<0,001
Doença cardíaca estrutural, n (%)	39 (7)	20 (5)	19 (11)	0,010
Apneia do sono, n (%)	44 (7)	32 (8)	12 (7)	0,63
História de doença tiroidiana, n (%)	121 (21)	77 (18)	44 (24)	0,21
Hipotiroidismo, n (%)	77 (13)	51 (12)	26 (14)	
Hipertiroidismo, n (%)	44 (7)	26 (6)	18 (10)	
Pacientes em uso de DAA classe IC ou Sotalol, n (%)	275 (46)	208 (51)	67 (37)	0,002
Pacientes em uso de amiodarona, n (%)	206 (35)	136 (33)	70 (39)	0,17
Escore CHA_2_DS_2_VASc (média ± DP)	1,8 ± 1,3	1,7 ± 1,3	2,0 ± 1,2	<0,001
Diâmetro AE (mm), (média ± DP)	44 ± 19	42 ± 6	49 ± 33	<0,001
FEVE, % (média ± DP)	57 ± 9	58 ± 8	54 ± 10	<0,001
Volume AE (angiotomografia), mL (média ± DP)	135 ± 51	123 ± 41	157 ± 61	<0,001
Índice de ablação (média ± DP)	457 ± 37	457 ± 40	459 ± 24	0,19
Presença de área de baixa voltagem, n (%)	154 (27)	58 (14)	96 (58)	<0,001

FA: fibrilação atrial; IMC: índice de massa corporal; AVC: acidente vascular cerebral; AE: atrial esquerdo; FEVE: fração de ejeção do ventrículo esquerdo.

Valores dos biomarcadores anteriores à ablação são descritos com detalhe na Tabela 2 . Os pacientes no grupo com FA persistente apresentaram níveis mais altos de TSH e BNP antes da ablação e comparação a pacientes com FA paroxística.

**Tabela 2:** Níveis dos biomarcadores nos pacientes com fibrilação atrial

	Todos os pacientes (n=593)	FA paroxística (n=412)	FA persistente (n=181)	Valor p
Clearance de creatinina, mL/min (média ± DP)	95 ± 41	94 ± 43	96 ± 36	0,64
Hemoglobina, g/dL (média ± DP)	14,0 ± 1,5	14,0 ± 1,5	14,1 ± 1,5	0,87
TSH, μUI/mL (média ± DP)	1,8 ± 1,7	1,6 ± 1,4	2,1 ± 2,2	<0,001
T_4_, ng/dL (mediana, Q1-Q3)	1,1 (1,0-1,2)	1,1 (1,0-1,3)	1,1 (1,0-1,2)	0,46
PCR, mg/dL (mediana, Q1-Q3)	0,2 (0,1-0,4)	0,2 (0,1-0,4)	0,2 (0,1-0,5)	0,17
BNP, pg/mL (mediana, Q1-Q3)	57 (25-120)	46 (22-10,2)	92 (47-167)	<0,001
Tn-us, ng/L (média±DP)	6 ± 20	5 ± 23	7 ± 13	0,37

FA: fibrilação atrial; TSH: hormônio tireoestimulante; T4: Tiroxina livre; PCR: Proteína C reativa; BNP: peptídeo natriurético cerebral; Tn-us: Troponina Ultrassensível.

### Biomarcadores e recorrência de arritmia

O tempo de acompanhamento médio foi de 24±6 meses. Em geral, não houve ocorrência de arritmia atrial nos três meses ( *blanking* ) após o procedimento em 76,4% dos pacientes (78,2% dos pacientes com FA paroxística e sem DAA, e 72,4% dos pacientes com FA persistente, sendo 50% sem uso de DAA).

**Figura 1:**
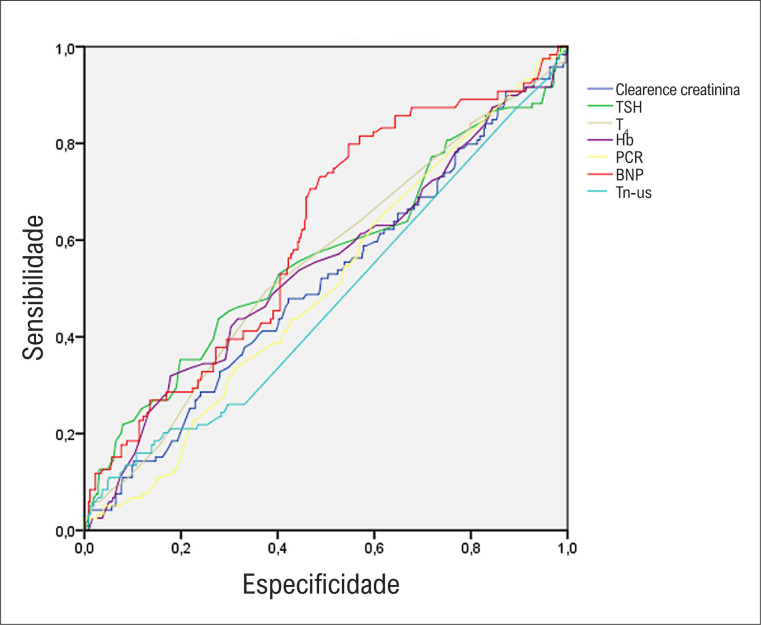
Curva ROC ilustrando o poder discriminatório de cada biomarcador. TSH: hormônio tireoestimulante; T4: Tiroxina livre; Hb: Hemoglobina; PCR: Proteína C reativa; BNP: peptídeo natriurético cerebral; Tn-us: Troponina Ultrassensível.

Os diferentes biomarcadores apresentaram poder preditivo modesto ou nulo para recorrência de arritmia quando usados isoladamente ( Figura 1 ): BNP pré-ablação [AUC 0,61, IC95% (0,56-0,65), p<0,001), TSH pré-ablação [AUC 0,58, IC95% (0,54-9,62), p=0,008); T_4_ livre pré-ablação [AUC 0,57, IC95% (0,53-0,61); p=0,-17); Hb pré-ablação [AUC 0,55; IC95% (0,50-0,59), p=0,11], Hs-cTnI pré-ablação [AUC 0,53, IC95% (0,49-0,57), p=0,19], Cl Cr pré-ablação [AUC 0.50, IC95% (0,46-0,54), p=0,97], PCR pré-ablação (AUC 0,50, IC95% (0,46-0,54), p=0,99). Ao considerar somente os biomarcadores com poder preditivo, os seguintes pontos de corte apresentaram melhores sensibilidade e especificidade combinadas e foram usados para análise subsequente: TSH de 1,8 μUI/mL (especificidade de 72%, sensibilidade de 47%, valor preditivo positivo de 81%) T_4_ livre de 1,1 ng/dL (especificidade de 63%, sensibilidade de 52%, valor preditivo positivo de 31%, valor preditivo negativo de 80%), e valor de BNP de 48pg/mL (especificidade de 50%, sensibilidade de 73%, valor preditivo positivo de 30%, valor preditivo negativo de 85%) (Figura S-2).

Na análise multivariada, hipertireoidismo, TSH > 1,8 μUI/mL e diâmetro atrial esquerdo foram preditores independentes de recorrência de arritmia, ao contrário de T4 livre e BNP ( Tabela 3 ).

**Tabela 3:** Preditores de recorrência de arritmia

	Todos os pacientes (n=593)	Sem recorrência de arritmia (n=453)	Com recorrência de arritmia (n=140)	Análise univariada		Análise multivariada	
				HR, (IC95%)	Valor p	HR, (95% CI)	Valor p
FA persistente, n (%)	181 (31)	131 (29)	50 (38)	1,31 (0,93-1,85)	0,13		
Sexo masculino, n (%)	345 (58)	260 (57)	85 (61)	1,24 (0,88-1,74)	0,22		
Idade, anos (média ±DP)	59 ± 12	59 ± 13	58 ± 11	0,99 (0,98-1,00)	0,15		
BMI, Kg/m^2^ (média ±DP)	27 ± 5	27 ± 4	28 ± 6	1,04 (0,99-1,08)	0,10		
Hipertensão, n (%)	357 (60)	266 (59)	91 (66)	1,22 (0,86-1,73)	0,27		
Diabetes mellitus, n (%)	376 (65)	284 (64)	92 (66)	1,19 (0,84-1,70)	0,33		
História de AVC, n (%)	27 (5)	24 (5)	3 (2)	0,54 (0,17-1,70)	0,29		
Insuficiência cardíaca congestiva, n (%)	135 (23)	97 (21)	38 (27)	1,27 (0,87-1,85)	0,22		
Doença cardíaca estrutural, n (%)	39 (7)	29 (6)	10 (7)	1,29 (0,68-2,45)	0,45		
Apneia do sono, n (%)	44 (7)	34 (8)	10 (7)	1,00 (0,53-1,91)	0,99		
História de doença tiroidiana							0,029
Hipotiroidismo	77 (13.0)	61 (13,5)	61 (13,5)	0,86 (0,51-1,46)	0,59	0,69 (0,35-1,34)	0,27
Hipertiroidismo, n (%)	44 (7.4)	17 (12,1)	17 (12,1)	1,74 (10,04-2,92)	0,034	2,05 (1,01-3,79)	0,022
TSH > 1,8 μUI/mL, n (%)	180 (33)	117 (28)	63 (40)	1,84 (1,31-2,56)	<0,001	1,82 (1,89-2,80)	0,006
T_4_ > 1,1 ng/dL, n (%)	225 (40)	155 (37)	70 (52)	1,46 (1,04-2,05)	0,029	1,12 (0,743-1,71)	0,60
BNP>48,3 pg/mL, n (%)	348 (59)	244 (54)	104 (74)	2,05 (1,40-3,00)	<0,001	1,28 (0,82-2,00)	0,29
FEVE, % (média ±DP)	57 ± 9	57 ± 9	57 ± 9	1,00 (0,94-1,02)	0,72		
Diâmetro AE (mm), (média ±DP)	44 ± 19	43 ± 7	48 ± 39	1,01 (1,01-1,02)	<0,001	1,01 (1,01-1,02)	<0,001
Volume AE (angiotomografia), mL (média ± DP)	135 ± 51	132 ± 48	145 ± 49	1,00 (1,00-1,01)	0,18		
Índice de ablação (média ± DP)	457 ± 37	458 ± 39	456 ± 30	1,00 (1,00-1,01)	0,88		
Presença de área de baixa voltagem, n (%)	154 (27)	115 (26)	39 (29)	1,01 (0,70-1,47)	0,95		

FA: fibrilação atrial; IMC: índice de massa corporal; AVC: acidente vascular cerebral; AE: atrial esquerdo; FEVE: fração de ejeção do ventrículo esquerdo; TSH: hormônio tireoestimulante; T_4_: Tiroxina livre; BNP: Peptídeo Natriurético Cerebral; Tn-us: Troponina Ultrassensível.

### Combinação de biomarcadores na predição de recorrência de arritmia

**Figura 2:**
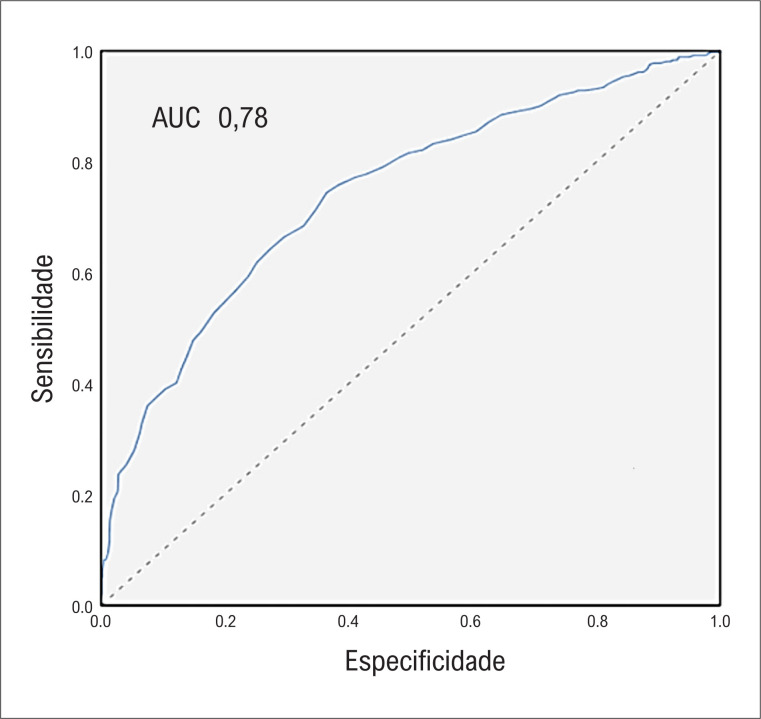
Curva ROC ilustrando o poder discriminatório de três biomarcadores (hormônio tireoestimulante; tiroxina livre; e peptídeo natriurético cerebral) combinados com uma AUC de 0,78, IC95% [0,74-0,83], p<0,001.

Os pacientes foram separados em diferentes grupos de acordo com o número de biomarcadores (0, 1, 2 ou 3) com valores anormais. Um número crescente de valores anormais de biomarcadores foi associado com menor sobrevida livre de recorrência de arritmia (87,1% para sem biomarcador vs. 83,5% para um vs. 75,1% para dois vs. 43,3% para três biomarcadores, p<0,001) (Figura Central). Após o ajuste quanto a outros fatores de confusão, os pacientes com três biomarcadores anormais apresentaram risco aumentado de recorrência de arritmia [HR=2,88 (IC95%, 1,39-5,17), p=0,003] ( Tabela 4 ). Além disso, a presença de três valores anormais de biomarcadores apresentou um bom poder preditivo para recorrência de arritmia (AUC 0,78; IC95% [0,74-0,83], p<0,001) ( Figura 2 ).

**Tabela 4:** Análise multivariada dos biomarcadores combinados para recorrência de arritmia

	Análise multivariada	
	HR, (IC95%)	Valor p
Diâmetro AE (mm)	1,01 (1,01-1,02)	<0,001
História de doença tiroidiana		0,050
Hipotiroidismo	0,71 (0,38-1,39)	0,32
Hipertiroidismo	1,88 (1,03-3,44)	0,040
Biomarcadores,0		<0,001
1	0,80 (0,43-1,50)	0,48
2	1,01 (0,54-1,89)	0,97
3	2,88 (1,39-5,17)	0,003

AE: atrial esquerdo.

### Sub-análise para pacientes com FA paroxística e FA persistente

Em relação aos pacientes com FA persistente, nenhum dos biomarcadores apresentou poder preditivo significativo. Para pacientes com FA paroxística, a combinação de três biomarcadores “anormais” (TSH, T_4_ livre e BNP) pôde prever a recorrência de arritmia (Tabelas S-1 e S-2).

## Discussão

Em nosso conhecimento, este é o primeiro estudo a avaliar o impacto de vários biomarcadores sobre a ablação da FA guiada pelo AI. Nossos achados sugerem que, individualmente, cada biomarcador pré-ablação tem nenhuma ou pouca capacidade de predizer a recorrência de arritmia (somente TSH foi independentemente associado com recorrência de arritmia durante o seguimento), mas a presença de múltiplos biomarcadores séricos anormais podem ajudar a predizer recorrência de arritmia após a ablação da FA guiada por AI.

Nos últimos anos, o manejo da FA melhorou substancialmente, sendo a ablação com cateter uma importante opção terapêutica. Contudo, apesar da melhoria dos desfechos após a ablação da FA, a recorrência de arritmia ainda não é incomum.^4^ Os biomarcadores séricos foram propostos como sendo de uso potencial para identificar pacientes em alto risco de recorrência por serem de fácil acesso em comparação a outros métodos, como tomografia computadorizada, ressonância magnética e exame por eletrofisiologia. Já foi demonstrado que os hormônios tireoidianos promovem um encurtamento da duração do potencial de ação e do período refratário, aumentando, assim, a automaticidade e atividade induzida nas veias pulmonares. Ainda, contribuem para o aumento da fibrose intersticial no átrio, estimulando o início ou a manutenção da FA.^25 – 29^ Essas mudanças fisiopatológicas provavelmente explicam a taxa mais alta de recidiva relacionada aos níveis de TSH em nosso estudo (o TSH foi um preditor independente de recorrência de arritmia), corroborando os achados observados por Morishima et al.,^29^ em que o TSH foi um preditor de arritmia atrial mesmo na faixa normal de TSH. Um dado importante foi que, ao contrário do previamente relatado,^30 , 31^ o valor anterior à ablação de TSH, mas não de T_4_ livre foi preditor independente de recorrência de FA, provavelmente porque os níveis de TSH reflitam com sensibilidade o *feedback* negativo do status tireoidiano.^32^ Nossos resultados sugerem que um melhor controle da função tiroidiana seja importante antes da ablação, embora não exista ainda evidência clara que corrobore terapia adicional com hormônio tiroidiano,^29^ e novos estudos sejam necessários para abordar esse tópico. No entanto, nossos dados sugerem que, no contexto de ablação guiada por AI, cada biomarcador tem pouco ou nenhum valor preditivo para recorrência de arritmia, incluindo o TSH.

Em relação ao BNP, a FA em si aumenta os níveis do peptídeo, o que está de acordo com nossos resultados, em que os pacientes com FA persistente apresentaram níveis mais altos de BNP antes da ablação que pacientes com FA paroxística. Entretanto, enquanto na FA paroxística, um valor de BNP de 48,3pg/mL na análise multivariada foi um preditor independente modesto de recorrência de arritmia (Tabela S-1), na FA persistente, nenhum dos biomarcadores, incluindo o BNP, apresentou significância estatística preditiva, mesmo quando combinados. A combinação de múltiplos biomarcadores (TSH, FT4 e BNP) pode ajudar a predizer recorrência de arritmia na FA paroxística, mas não na FA persistente. Quando esses três biomarcadores eram “anormais”, a recorrência de arritmia foi quase três vezes mais alta que a ausência de biomarcadores “anormais” na FA paroxística. Com esses resultados, podemos levantar a hipótese de que, diferentemente da FA paroxística, na FA persistente, o remodelamento atrial, causado por estágios avançados da doença e outros fatores não testados em nosso estudo, podem exercer um papel significativo na recorrência de arritmia, o que pode explicar a incapacidade desses biomarcadores séricos em predizer a recorrência de FA.

Foi demonstrado que o software AI estima a profundidade da lesão, permitindo, assim, que a ablação seja ajustada às diferentes espessuras de parede do átrio esquerdo, o que poderia, em teoria, reduzir a capacidade de alguns biomarcadores em predizer recorrências.^5 , 33 – 35^ Portanto, principalmente na FA paroxística, o uso de múltiplos biomarcadores (TSH, T_4_ livre e BNP) combinados pode ser de grande interesse.

Uma vez que esse simples escore de risco tem um poder preditivo razoavelmente bom, os médicos conseguem facilmente avaliar esses biomarcadores antes da ablação da FA guiada pelo AI, quando o remodelamento atrial ainda não está estabelecido. Na presença de três biomarcadores “anormais”, dado o risco mais alto de recorrência, os médicos devem ser mais cuidadosos em explicar o risco e o benefício da ablação, e considerar tratamento individualizado antes da ablação por cateter. Por exemplo, a terapia com hormônio tiroidiano foi proposta por alguns autores na presença de hipertiroidismo subclínico e FA; perindopril diminui o nível de angiotensina II e foi associado com uma redução de recorrência de FA após a ablação por cateter; e terapias para prevenção da degradação do peptídeo natriurético também foram propostas.^11 , 36 – 38^ Embora essa informação seja nova, mais estudos são necessários para confirmar esses resultados e avaliar o papel de novos alvos para intervenção farmacológica antes da ablação por cateter.

Há várias limitações em nosso estudo. Primeiro, o nível de biomarcadores séricos pode ser afetado por doenças cardíacas e não cardíacas. Segundo, os pontos de corte usados para definir o biomarcador como “normal” e “anormal” em nosso estudo podem variar de acordo com as características dos pacientes e faixas de valores laboratoriais. Terceiro, o uso de amiodarona antes da ablação pode ter afetado os valores de TSH e T_4_ livre. No entanto, nosso objetivo foi apresentar uma avaliação real desses biomarcadores na prática clínica, em que uma porcentagem considerável dos pacientes é tratada com amiodarona. Contudo, mesmo excluindo os pacientes com amiodarona, pacientes com três biomarcadores “anormais” ainda mantêm um risco mais alto de recorrência de arritmia em comparação aos pacientes sem nenhum biomarcador. Quarto, é provável que a taxa de recorrência relatada neste estudo esteja subestimada, dado que o monitoramento pós-ablação foi somente intermitente. O uso de monitores cardíacos implantáveis permitiria a documentação do real peso da arritmia. Porém, uma vez que o monitoramento foi idêntico em todos os pacientes, essa limitação não teve impacto sobre nossos resultados principais.

## Conclusão

Valores anormais de TSH, T_4_ livre e BNP, quando combinados, podem ajudar a predizer a recorrência de arritmia após a ablação guiada por AI. Mais estudos são necessários para esclarecer se é necessário identificar valores ótimos desses biomarcadores antes da ablação.

## *Material suplementar

Para informação adicional, por favor, clique aqui
