# Supplementary material for: Os Biomarcadores podem ser Utilizados para Prever Recorrência de Arritmia após a Ablação de Fibrilação Atrial Guiada pelo Ablation-Index ?
Source: Arq Bras Cardiol. 2024 Apr 15;121(4):e20230544. [Article in Portuguese] doi: 10.36660/abc.20230544 (PMC11081145; doi:10.36660/abc.20230544)
Supplement: Translation

# Translation: Can Pre-Ablation Biomarkers Be Used to Predict Arrhythmia Recurrence after Ablation Index-Guided Atrial Fibrillation Ablation?

**DOI:** 10.36660/abc.20230544i

**Authors:** Andreia Palma, Pedro A. Sousa, Carolina Saleiro, Sérgio Barra, Natália António, Luis Adão, João Primo, Ana Lebreiro, Paulo Fonseca, Luís Elvas, Lino Gonçalves

**Affiliations:** 1 Coimbra Portugal Hospital Pediátrico de Coimbra, Coimbra – Portugal; 2 Coimbra Portugal Departamento de Eletrofisiologia e Estimulação – Centro Hospitalar e Universidade de Coimbra, Coimbra – Portugal; 3 Vila Nova de Gaia Portugal Hospital da Luz Arrábida, Vila Nova de Gaia – Portugal; 4 Porto Portugal Departamento de Cardiologia do Centro Hospitalar e Universitário São João, Porto – Portugal; 5 Vila Nova de Gaia Portugal Departamento de Cardiologia – Vila Nova de Gaia e Espinho Hospital, Vila Nova de Gaia – Portugal

**Keywords:** Biomarkers, Catheter Ablation, Atrial Fibrillation, Cardiac Arrhythmias

## Abstract

**Background:**

Ablation Index (AI) software has allowed better atrial fibrillation (AF) ablation results, but recurrence rates remain significant. Specific serum biomarkers have been associated with this recurrence.

**Objectives:**

To evaluate whether certain biomarkers could be used (either individually or combined) to predict arrhythmia recurrence after AI-guided AF ablation.

**Methods:**

Prospective multicenter observational study of consecutive patients referred for AF ablation from January 2018 to March 2021. Hemoglobin, brain natriuretic peptide (BNP), C-reactive protein, high sensitivity cardiac troponin I, creatinine clearance, thyroid-stimulating hormone (TSH) and free thyroxine (FT_4_) were assessed for their ability to predict arrhythmia recurrence during follow-up. Statistical significance was accepted for p values of<0.05.

**Results:**

A total of 593 patients were included - 412 patients with paroxysmal AF and 181 with persistent AF. After a mean follow-up of 24±6 months, overall single-procedure freedom from atrial arrhythmia was 76.4%. Individually, all biomarkers had no or only modest predictive power for recurrence. However, a TSH value >1.8 μUI/mL (HR=1.82 [95% CI, 1.89-2.80], p=0.006) was an independent predictor of arrhythmia recurrence. When assessing TSH, FT_4_and BNP values in combination, each additional “abnormal” biomarker value was associated with a lower freedom from arrhythmia recurrence (87.1 % for no biomarker vs. 83.5% for one vs. 75.1% for two vs. 43.3% for three biomarkers, p<0.001). Patients with three “abnormal” biomarkers had a threefold higher risk of AF recurrence compared with no “abnormal” biomarker (HR=2.88 [95% CI, 1.39-5.17], p=0.003).

**Conclusions:**

When used in combination, abnormal TSH, FT_4_and BNP values can be a useful tool for predicting arrhythmia recurrence after AI-guided AF ablation.

## Introduction

Atrial fibrillation (AF) is the most common sustained cardiac arrhythmia and cause of high health and social care costs due to recurrent use of health services for symptom management and associated morbidity.^1 , 2^The AF prevalence increases with age, ranging from 0.5% among individuals aged 50 to 59 years to 8.8% among individuals aged 80 to 89 years.^3^Radiofrequency (RF) catheter ablation (CA) has emerged as a therapeutic option for AF, but despite recent advances, recurrence rates are considerably high.^4^Recently, a new software entitled “Ablation Index” (AI) (Biosense Webster), which incorporates contact-force (CF), time and power in a weighted formula, has been associated with lower pulmonary vein reconnection and higher freedom from atrial arrhythmias, ranging from 78% to more than 90%.^5 – 9^

Identifying a higher risk of arrhythmia recurrence may help physicians to select patients for ablation, inform them about the risk-benefit ratio and select the optimal ablation strategy. Several biomarkers have been associated with atrial arrhythmia recurrence following AF ablation, but results have varied substantially across studies and the predictive power of such biomarkers has been low.^10 – 18^Most of these studies have evaluated the impact of biomarkers individually rather than including them in a multiparametric score. Also, with the exception of one study,^19^no biomarker was evaluated in the context of ablation guided by a software developed to predict transmural lesions.

**Figure:** Kaplan-Meier curves for freedom from atrial arrhythmia recurrence, according to the number of “abnormal” biomarkers present (87.1 % for no biomarker vs. 83.5% for one vs. 75.1% for two biomarkers vs. 43.3% for three biomarkers, p<0.001).

This study aims to identify pre-ablation serum biomarkers associated with arrhythmia recurrence, in the setting of AF ablation guided by the AI software.

## Methods

### Study design and setting

Prospective multicenter observational study of consecutive patients referred for AF ablation from January 2018 to March 2021. Patients with paroxysmal and persistent AF, referred for CA, were submitted to a specific ablation protocol. Baseline clinical data and ablation parameters were obtained from hospital databases. Values of pre-ablation serum biomarkers were obtained, and we evaluated whether they were associated with arrhythmia recurrence during follow-up.

All patients provided written informed consent and the study was approved by the local institutional ethics committee.

### Patient eligibility criteria

Patients were eligible for inclusion in the study if they met the following inclusion criteria: 1) paroxysmal, persistent or long-standing persistent AF patients aged ≥18 years, refractory to or intolerant of anti-arrhythmic drug (AAD) therapy; 2) submitted to RF ablation with an irrigated-tip contact force-sensing catheter guided by the AI software. Paroxysmal AF was defined as AF terminating spontaneously or cardioverted within seven days; persistent AF was defined as AF sustained beyond seven days or cardioverted after seven or more days; and long-standing persistent AF was defined as a one-year continuous AF with a rhythm control strategy, according to the 2020 European Society of Cardiology (ESC) Guidelines for the Management of Atrial Fibrillation developed in collaboration with the European Heart Rhythm Association (EACTS).^20^

Exclusion criteria were the following: previous history of AF ablation or clinically apparent acute coronary syndrome, contraindication to anticoagulation and presence of intracardiac thrombus detected prior to the ablation procedure. Patients with overt hyperthyroidism or overt hypothyroidism were also considered as having a contraindication for CA.

Known history of thyroid disease was defined as previous history of thyroiditis, thyroidectomy, or ongoing medical treatment for hypothyroidism or hyperthyroidism, independently of current levels of free thyroxine (FT_4_).

### Ablation Procedure

Details of the periprocedural management and the tailored techniques for paroxysmal and persistent AF ablation conducted in our institution have been previously published^21 – 23^and are described in detail in the Supplement Data.

### Biomarkers measurement

All serum biomarkers – hemoglobin (Hb), brain natriuretic peptide (BNP), C-reactive protein (CRP), high sensitivity cardiac Troponin I (Hs-cTnI), creatinine clearance (Cr Cl), thyroid-stimulating hormone (TSH) and FT_4_– were measured up to 18 hours before the procedure (irrespective of the heart rhythm), with the patient lying in the supine position, according to local protocols. For the measurement of the serum Hs-cTnI (expressed in ng/L), the plasma was separated by centrifugation at 3500 rpm for 15 min and measured right away. Hs-cTnI levels were analyzed using an Abbott Troponin I, Alinity^®^diagnostics assay. Cutoff for 99^th^percentile of Hs-cTnI was 16 ng/L. Plasma levels of CRP were measured using an immunoassay on latex (immunoturbidimetry) assay (Alinity c CRP Vario, Abbott Diagnostics). The reference values for CRP are below 0.5mg/dL; with the lower limit of detection for this assay being 0.1mg/dL, and the highest being 48 mg/dL. BNP levels (pg/mL) were measured with an autoanalyzer (Alinity, Abbott Diagnostics) using chemiluminescent microparticle immunoassay. The normal cut-off value was < 100 pg/mL. Serum TSH and FT_4_were assessed with a chemiluminescent microparticle immunoassay (Alinity, Abbott Diagnostics). Laboratory reference ranges for FT_4_and TSH were 0.7 to 1.5 ng/dL and 0.4 to 4.0 μUI/mL, respectively. Serum creatinine was assessed with a commercial immunoassay on latex (Alinity, Abbott Diagnostics). Creatinine levels (mg/dL) were considered normal between 0.55 to 1.02 mg/dL (female) and 0.72 to 1.18 mg/dL (male). The Cr Cl was calculated using the Cockcroft-Gault equation. Hb levels (expressed as g/dL) were measured by photometry using an automated hematology system (Sysmex XN-9000, Sysmex) and the SLS-Hgb method (cyanide-free sodium lauryl sulphate). In male gender, Hb levels between 13.5 to 17.5 g/dL (18 to 49 years), 12.0 to 15.6 g/dL (49 to 65 years) and 11.8 to 15.8 g/dL (>65 years) were considered normal. In female gender, the cut-off values for Hb were 12.0 to 16.0 g/dL (18 to 49 years), 12.0 to 15.6 g/dL (49 to 65 years) and 11.8 to 15.8 g/dL (if >65 years).

### Study endpoints

The primary objective was to assess whether serum biomarkers, either alone or combined, can be used to predict arrhythmia recurrence after AI-guided AF ablation. Arrhythmia recurrence was defined as the documentation of at least 30 seconds of any sustained atrial arrhythmia after a 3-month blanking period, irrespective of symptoms.^24^

### Follow-up

After the index procedure, patients were followed for a minimum of 12 months. Patients were evaluated before discharge, as well as at three, six, 12, 18 and 24 months after the procedure. Transthoracic echocardiography and 24-hour Holter monitoring were performed before discharge. Information collected during follow-up included a 12-lead electrocardiogram and a 24-hour Holter in each appointment. A seven-day Holter monitoring was performed at least once a year. At discharge, AAD were interrupted in patients with paroxysmal AF (except for beta-blockers, which were allowed to be continued). In patients with persistent or long-standing persistent AF, AAD prescription was left at the physician's discretion. The first three months post-procedure were considered as a blanking period, and recurrences during this period were not considered. The anticoagulation strategy after the first 3 months was based on the CHA2DS2Vasc score.

### Statistical analysis

Statistical analysis was performed using IBM SPSS Statistics version 25 (IBM, Armonk, New York) software and MedCalc Software Ltd. Categorical variables were expressed in frequencies and percentages; continuous variables with and without normal distribution were expressed as mean ± standard deviation and median and interquartile range (IQR), respectively. The X^2^test was used to assess differences between categorical variables and the unpaired Student's t-test and the Mann-Whitney-Wilcoxon test were used to compare continuous variables with and without normal distribution, respectively. The Kolmogorov-Smirnov test was used to test for normality of distribution of continuous variables. The area under the ROC curve (AUC) was used to test the discriminative performance of each biomarker, or their combination, in the prediction of arrhythmia recurrence. For each predictor, the value with best sensitivity and specificity was defined according to the Youden Index. This value was used to dichotomise serum biomarkers as “normal” or “abnormal” and was further used as a dichotomous variable in survival analysis. Kaplan-Meier curves were created to illustrate arrhythmia-free survival according to the different combinations of biomarkers. A Cox proportional-hazards model with time-dependent covariates for changing the combination of biomarkers and AF recurrence was built to evaluate the independent effect of these combinations on outcomes. All demographic, clinical and laboratorial variables considered to have impact on recurrence were tested in a univariable analysis. Those variables which reached statistical significance were further included in multivariable analysis model. A sub-analysis for paroxysmal AF and persistent AF patients was carried out as well. Statistical significance was accepted for p values < 0.05.

## Results

Of the initial 705 patients submitted to AF ablation during the enrollment period of the study, 98 were excluded due to previous history of AF ablation, and 14 patients were lost to follow-up (Figure S-1). The final sample included 593 patients, corresponding to 412 patients with paroxysmal AF and 181 patients with persistent and long-standing persistent AF. Several differences in baseline characteristics were found between paroxysmal AF patients and the persistent AF population ( Table 1 ). There were no differences regarding the existence of known thyroid disease or previous treatment with amiodarone.

**Table 1:** Baseline characteristics of patients with atrial fibrillation

	All Patients (n=593)	Paroxysmal AF (n=412)	Persistent AF (n=181)	p value
Male gender, n (%)	(n=593)	234 (57)	111 (61)	0.30
Age, years (mean ±SD)	(n=412)	58 ± 12	60 ± 13	0.07
BMI, Kg/m2 (mean ±SD)	(N=181)	27 ± 5	27 ± 4	0.59
Hypertension, n (%)	235 (40)	244 (59)	113 (62)	0.48
Diabetes mellitus, n (%)	376 (65)	268 (67)	108 (61)	0.16
Stroke history, n (%)	27 (5)	18 (4)	9 (5)	0.75
Congestive heart failure, n (%)	132 (23)	60 (15)	71 (41)	<0.001
Structural heart disease, n (%)	39 (7)	20 (5)	19 (11)	0.010
Sleep apnea, n (%)	44 (7)	32 (8)	12 (7)	0.63
History of thyroid disease, n (%)	121 (21)	77 (18)	44 (24)	0.21
Hypothyroidism, n (%)	77 (13)	51 (12)	26 (14)	
Hyperthyroidism, n (%)	44 (7)	26 (6)	18 (10)	
Patients under class **IC** AAD or Sotalol, n (%)	275 (46)	208 (51)	67 (37)	0.002
Patients under Amiodarone treatment, n (%)	206 (35)	136 (33)	70 (39)	0.17
CHA_2_DS_2_VASc score (mean ±SD)	1.8 ± 1.3	1.7 ± 1.3	2.0 ± 1.2	<0.001
LA diameter (mm), (mean ±SD)	44 ± 19	42 ± 6	49 ± 33	<0.001
LVEF, % (mean ±SD)	57 ± 9	58 ± 8	54 ± 10	<0.001
CCTA LA volume, mL (mean ±SD)	135 ± 51	123 ± 41	157 ± 61	<0.001
Ablation index (mean ±SD)	457 ± 37	457 ± 40	459 ± 24	0.19
Presence of low voltage area, n (%)	154 (27)	58 (14)	96 (58)	<0.001

AF: atrial fibrillation; BMI: body mass index; AAD: anti-arrhythmic drug; CCTA: cardiac computed tomography angiography; LA: left atrium; LVEF: left ventricle ejection fraction.

Pre-ablation biomarker values are detailed in Table 2 . Patients in the persistent AF group had higher TSH and BNP levels before ablation compared to patients with paroxysmal AF.

**Table 2:** Biomarkers levels in patients with atrial fibrillation

	All Patients (n=593)	Paroxysmal AF (n=412)	Persistent AF (n=181)	p value
Clearance of creatinine, ml/min (mean ±SD)	95 ± 41	94 ± 43	96 ± 36	0.64
Hemoglobin, g/dL (mean ±SD)	14.0 ± 1.5	14.0 ± 1.5	14.1 ± 1.5	0.87
TSH, μUI/mL (mean±SD)	1.8 ± 1.7	1.6 ± 1.4	2.1 ± 2.2	<0.001
FT_4_, ng/dL (median, Q1-Q3)	1.1 (1.0-1.2)	1.1 (1.0-1.3)	1.1 (1.0-1.2)	0.46
CRP, mg/dL (median, Q1-Q3)	0.2 (0.1-0.4)	0.2 (0.1-0.4)	0.2 (0.1-0.5)	0.17
BNP, pg/mL (median, Q1-Q3)	57 (25-120)	46 (22-10.2)	92 (47-167)	<0.001
Hs-cTnI, ng/L (mean±SD)	6 ± 20	5 ± 23	7 ± 13	0.37

AF: atrial fibrillation; TSH: thyroid-stimulating hormone; BNP: brain natriuretic peptide.

### Biomarkers and arrhythmia recurrence

Mean follow-up was 24±6 months. Overall, single-procedure freedom from atrial arrhythmia after the 3-month blanking period was 76.4% (78.2% in paroxysmal AF, off-AAD and 72.4% in persistent AF, with 50% off-AAD).

The different biomarkers had no or only modest predictive power for arrhythmia recurrence when used alone ( Figure 1 ): BNP pre-ablation (AUC 0.61, 95% CI [0.56-0.65], p<0.001), TSH pre-ablation (AUC 0.58, 95% CI [0.54-0.62], p=0.008), FT_4_pre-ablation (AUC 0.57, 95% CI [0.53-0.61], p=0.017), Hb pre-ablation (AUC 0.55, 95% CI [0.50-0.59], p=0.11), Hs-cTnI pre-ablation (AUC 0.53, 95% CI [0.49-0.57], p=0.19), Cl Cr pre-ablation (AUC 0.50, 95% CI [0.46-0.54], p=0.97), CRP pre-ablation (AUC 0.50, 95% CI [0.46-0.54], p=0.99). When considering only biomarkers with predictive power of AF recurrence, the following cut-offs had the best combined sensitivity and specificity and were used for subsequent analysis: TSH value of 1.8 μUI/mL (72% specificity, 47% sensitivity, positive predictive value 35%, negative predictive value 81%), FT_4_value of 1.1 ng/dL (specificity 63%, sensitivity 52%, positive predictive value 31%, negative predictive value 80%) and BNP value of 48 pg/mL (specificity 50%, sensitivity 73%, positive predictive value 30%, negative predictive value 85%) (Figure S-2).

**Figure 1:** ROC curve illustrating the discriminative power of each biomarker. TSH: thyroid-stimulating hormone; T4: Free thyroxine; Hb: Hemoglobin; CRP: C-reactive protein; BNP: brain natriuretic peptide; Tn-us: Ultrasensitive Troponin.

In multivariate analysis, hyperthyroidism, TSH value >1.8 μUI/mL and LA diameter were independent predictors of arrhythmia recurrence, while FT4 and BNP were not ( Table 3 ).

**Table 3:** Predictors of arrhythmia recurrence

	All Patients (n=593)	Without arrhythmia recurrence (n=453)	With arrhythmia recurrence (n=140)	Univariate analysis		Multivariate analysis	
				HR, (95% CI)	p value	HR, (95% CI)	p value
Persistent AF, n (%)	181 (31)	131 (29)	50 (38)	1.31 (0.93-1.85)	0.13		
Male gender, n (%)	345 (58)	260 (57)	85 (61)	1.24 (0.88-1.74)	0.22		
Age, years (mean ±SD)	59 ± 12	59 ± 13	58 ± 11	0.99 (0.98-1.00)	0.15		
BMI, Kg/m^2^(mean ±SD)	27 ± 5	27 ± 4	28 ± 6	1.04 (0.99-1.08)	0.10		
Hypertension, n (%)	357 (60)	266 (59)	91 (66)	1.22 (0.86-1.73)	0.27		
Diabetes mellitus, n (%)	376 (65)	284 (64)	92 (66)	1.19 (0.84-1.70)	0.33		
Stroke history, n (%)	27 (5)	24 (5)	3 (2)	0.54 (0.17-1.70)	0.29		
Congestive heart failure, n (%)	135 (23)	97 (21)	38 (27)	1.27 (0.87-1.85)	0.22		
Structural Heart Disease, n (%)	39 (7)	29 (6)	10 (7)	1.29 (0.68-2.45)	0.45		
Sleep apnea, n (%)	44 (7)	34 (8)	10 (7)	1.00 (0.53-1.91)	0.99		
History of thyroid disease,							0.029
Hypothyroidism	77 (13.0)	61 (13.5)	61 (13.5)	0.86 (0.51-1.46)	0.59	0.69 (0.35-1.34)	0.27
Hyperthyroidism n (%)	44 (7.4)	17 (12.1)	17 (12.1)	1.74 (10.04-2.92)	0.034	2.05 (1.01-3.79)	0.022
TSH > 1.8 μUI/mL, n (%)	180 (33)	117 (28)	63 (40)	1.84 (1.31-2.56)	<0.001	1.82 (1.89-2.80)	0.006
FT_4_> 1.1 ng/dL, n (%)	225 (40)	155 (37)	70 (52)	1.46 (1.04-2.05)	0.029	1.12 (0.743-1.71)	0.60
BNP>48.3 pg/mL, n (%)	348 (59)	244 (54)	104 (74)	2.05 (1.40-3.00)	<0.001	1.28 (0.82-2.00)	0.29
LVEF, % (mean ±SD)	57 ± 9	57 ± 9	57 ± 9	1.00 (0.94-1.02)	0.72		
LA diameter (mm), (mean ±SD)	44 ± 19	43 ± 7	48 ± 39	1.01 (1.01-1.02)	<0.001	1.01 (1.01-1.02)	<0.001
CCTA LA volume, mL (mean ±SD)	135 ± 51	132 ± 48	145 ± 49	1.00 (1.00-1.01)	0.18		
Mean Ablation index (mean ±SD)	457 ± 37	458 ± 39	456 ± 30	1.00 (1.00-1.01)	0.88		
Presence of low voltage area, n (%)	154 (27)	115 (26)	39 (29)	1.01 (0.70-1.47)	0.95		

AF: atrial fibrillation; BMI: body mass index; CCTA: cardiac computed tomography angiography; LA: left atrium; LVEF: left ventricle ejection fraction.

### Combining multiple biomarkers for predicting arrhythmia recurrence

Patients were split into different groups according to whether they had 0, 1, 2 or 3 abnormal biomarker values. An increasing number of “abnormal” biomarker values was associated with lower freedom from arrhythmia recurrence (87.1% for no biomarker vs. 83.5% for one vs. 75.1% for two vs. 43.3% for three biomarkers, p<0.001) (Central Illustration). After adjusting for other confounders, patients with three abnormal biomarkers had increased risk of arrhythmia recurrence (HR=2.88 [95% CI, 1.39-5.17], p=0.003) ( Table 4 ). Moreover, the presence of three abnormal biomarker values had a good predictive power for arrhythmia recurrence (AUC 0.78, 95% CI [0.74-0.83], p<0.001) ( Figure 2 ).

**Table 4:** Multivariate analysis of combined biomarkers for arrhythmia recurrence

	Multivariate analysis	
	HR, (95% CI)	p value
LA diameter (mm)	1.01 (1.01-1.02)	<0.001
History of thyroid disease,		0.050
Hypothyroidism	0.71 (0.38-1.39)	0.32
Hyperthyroidism	1.88 (1.03-3.44)	0.040
Biomarcadores,0		<0.001
1	0.80 (0.43-1.50)	0.48
2	1.01 (0.54-1.89)	0.97
3	2.88 (1.39-5.17)	0.003

LA: left atrial.

**Figure 2:** ROC curve illustrating the discriminative power of the three biomarker values (TSH, FT4 and BNP) combined with an AUC 0.78, 95% CI [0.74-0.83], p<0.001.

### Sub-analysis for paroxysmal and persistent AF patients

With respect to persistent AF patients, none of the biomarkers had significant predictive power. For paroxysmal AF patients, the combination of three “abnormal” biomarkers (TSH, FT_4_and BNP) could predict arrhythmia recurrence (Table S-1 and Table S-2).

## Discussion

To our knowledge, this is the first study assessing the impact of several serum biomarkers on AI-guided AF ablation. Our findings suggest that, individually, each pre-ablation serum biomarker has no or only modest ability in predicting arrhythmia recurrence (only TSH was independently associated with arrhythmia recurrence during follow-up), but the presence of multiple “abnormal” serum biomarkers can help predict arrhythmia recurrence after AI-guided AF ablation.

In recent years, AF management has substantially improved, with catheter ablation being an important therapeutic option. However, despite improved outcomes after AF ablation, arrhythmia recurrence is still not uncommon.^4^Serum biomarkers have been proposed as being of potential use to identify patients at higher risk of recurrence since they are very easily accessible compared with other methods, such as computed tomography, magnetic resonance imaging and electrophysiologic study. It has already been demonstrated that thyroid hormones promote shortening of the action potential duration and refractory period. Consequently, they enhance automaticity and triggered activity in the pulmonary veins and also increased interstitial fibrosis in the atrium, which can serve as drivers for the beginning or maintenance of AF.^25 – 29^These pathophysiological changes can probably explain the higher relapse rate related to TSH levels in our study (TSH was an independent predictor of arrhythmia recurrence), corroborating the findings observed by Morishima et al.,^29^where TSH was a predictor of atrial arrhythmia even at normal TSH range. Importantly, contrary to what has been previously reported,^30 , 31^pre-ablation value of TSH but not FT_4_independently predicted AF recurrence, probably because TSH levels sensitively reflect the negative feedback of thyroid status.^32^Our results suggest that a better control of thyroid function is important before ablation, although there is still no clear evidence supporting additional thyroid hormone therapy,^29^and new studies are required to address this topic. Nevertheless, our data suggests that, in the setting of AI-guided ablation, each biomarker has no or only modest predictive value for arrhythmia recurrence, including TSH.

Regarding the BNP value, AF itself increases BNP levels, which is in line with our results, where persistent AF patients had higher BNP levels before ablation than patients with paroxysmal AF. However, whereas in paroxysmal AF, a BNP value of 48.3 pg/ml in multivariate analysis was a modest independent predictor of arrhythmia recurrence (Table S-1), in persistent AF, none of the biomarkers, including BNP, had predictive statistical significance, even in combination. Interestingly, the combination of multiple biomarkers (TSH, FT4 and BNP) can help predict arrhythmia recurrence in paroxysmal AF but not in persistent AF. When all these three biomarkers were “abnormal”, arrhythmia recurrence was almost threefold higher compared with the absence of “abnormal” biomarkers in paroxysmal AF. With these results, we can hypothesize that, unlike paroxysmal AF, in persistent AF, atrial remodeling caused by advanced stages of the disease, and other factors not tested in our study, may play a significant role in arrhythmia recurrence, which may explain the inability of these serum biomarkers to predict recurrence.

The AI software has been shown to estimate lesion depth, thus allowing ablation to be tailored to the thickness of the different left atrial walls, which could, in theory, reduce the ability of some biomarkers to predict recurrences.^5 , 33 – 35^Thus, mainly in paroxysmal AF, the use of multiple biomarkers (TSH, FT_4_and BNP) combined may be of greater interest.

Since this simple risk score has reasonably good predictive power, physicians can easily assess these biomarkers prior to AI-guided AF ablation to predict the outcome in the early stages of the disease, when atrial remodeling is not yet established. In the presence of three “abnormal” biomarkers, given the higher risk of recurrence, physicians should more carefully explain the risk-benefit ratio of ablation, and consider individualized treatment before catheter ablation. For example, thyroid hormone therapy has been proposed by some authors in the presence of subclinical hyperthyroidism and AF; perindopril decreases the level of angiotensin-II and has been associated with a reduction of AF recurrence after catheter ablation; and therapies preventing natriuretic peptide degradation, like angiotensin receptor neprilysin inhibitor have also been proposed.^11 , 36 – 38^Although this information is novel, further studies are required to confirm these findings and assess the role of novel targets for pharmacological intervention before catheter ablation.

We acknowledge several limitations in our work. First, the level of serum biomarkers may be affected by cardiac and non-cardiac diseases. Second, the cut-off values used to define the biomarker as “normal” or “abnormal” in our study could vary according to patients’ characteristics and laboratory range values. Third, the use of amiodarone prior to ablation might have affected the TSH and FT4 values. However, our goal was to provide a real-world assessment of these biomarkers in the clinical practice, where a considerable percentage of patients are treated with amiodarone. Nevertheless, even excluding patients with amiodarone, patients with three “abnormal” biomarkers still maintain a higher risk of arrhythmia recurrence compared to patients without any biomarker. Fourth, it is likely that the recurrence rate reported in this study is underestimated, given that the post-ablation monitoring was only intermittent. The use of implantable loop recorders would have allowed documentation of the true arrhythmia burden. However, as monitoring was identical in all patients, this limitation had no impact on our main findings.

## Conclusion

Abnormal TSH, FT_4_and BNP values, when used in combination, may help predict arrhythmia recurrence after AI-guided ablation. Further studies should clarify if optimal biomarker values should be identified prior to ablation.
